# Integrated online formative assessments in the biomedical sciences for medical students: benefits for learning

**DOI:** 10.1186/1472-6920-8-52

**Published:** 2008-11-25

**Authors:** Gary M Velan, Philip Jones, H Patrick McNeil, Rakesh K Kumar

**Affiliations:** 1 Department of Pathology, School of Medical Sciences, Faculty of Medicine, The University of New South Wales, Sydney 2052, Australia; 2 Office of Medical Education, Faculty of Medicine, The University of New South Wales, Sydney 2052, Australia; 3 South West Sydney Clinical School, Faculty of Medicine, The University of New South Wales, Sydney 2052, Australia

## Abstract

**Background:**

Online formative assessments have a sound theoretical basis, and are prevalent and popular in higher education settings, but data to establish their educational benefits are lacking. This study attempts to determine whether participation and performance in integrated online formative assessments in the biomedical sciences has measurable effects on learning by junior medical students.

**Methods:**

Students enrolled in Phase 1 (Years 1 and 2) of an undergraduate Medicine program were studied over two consecutive years, 2006 and 2007. In seven consecutive courses, end-of-course (EOC) summative examination marks were analysed with respect to the effect of participation and performance in voluntary online formative assessments. Online evaluation surveys were utilized to gather students' perceptions regarding online formative assessments.

**Results:**

Students rated online assessments highly on all measures. Participation in formative assessments had a statistically significant positive relationship with EOC marks in all courses. The mean difference in EOC marks for those who participated in formative assessments ranged from 6.3% (95% confidence intervals 1.6 to 11.0; p = 0.009) in Course 5 to 3.2% (0.2 to 6.2; p = 0.037) in Course 2. For all courses, performance in formative assessments correlated significantly with EOC marks (p < 0.001 for each course). The variance in EOC marks that could be explained by performance in the formative assessments ranged from 21.8% in Course 6 to 4.1% in Course 7.

**Conclusion:**

The results support the contention that well designed formative assessments can have significant positive effects on learning. There is untapped potential for use of formative assessments to assist learning by medical students and postgraduate medical trainees.

## Background

Assessment has sufficiently powerful effects on learning to be the *de facto *curriculum [1]. This includes not only what is learnt, but also students' approaches to learning [2-4]. Formative assessments are designed for the purpose of giving feedback on performance and suggestions for improvement, and are intended to promote students' learning [5,6]. Formative assessments that provide timely, relevant and supportive feedback (not just grades) can contribute to improved learning outcomes [7]. In contrast, summative assessments are predominantly utilized for grading and certification at the end of a period of study, often without providing feedback to students on their performance. Indeed, one of the major weaknesses of most modern higher education programs, as evidenced by course evaluation surveys, is failure to provide adequate feedback to students on their learning [8]. It should be noted that the provision of diagnostic and remedial feedback has been found to be one of the most potent influences on student achievement [9]. If the purpose of assessment is to foster better learning outcomes, it could be argued that formative assessment is the most important assessment practice [10].

Paper-based formative assessments have a number of limitations [11]: students must be gathered together and invigilated; individualized feedback is time-consuming, and might not be feasible with large class sizes [12]; and analysis of question reliability and validity can be tedious. In contrast, Web-based formative assessments offer clear advantages for students: immediacy of feedback; flexibility in time and place of undertaking the assessment; feedback can provide links to learning resources, thereby providing motivation to study; opportunity for repetition; and interactivity [11]. Furthermore, a comparative study has reported that online formative assessments might be of greater benefit for learning than paper-based equivalents [13]. These are persuasive arguments for moving from paper-based to online formative assessments.

Web-based formative assessments also support equity and inclusiveness by allowing students to attempt each assessment anonymously on multiple occasions, at any time, and from virtually anywhere. This permits students with family responsibilities and work commitments to access the assessments at times that are most convenient for them. Many students, particularly those from culturally and linguistically diverse backgrounds, fear embarrassment if found to be in error, which might inhibit their propensity to clarify misconceptions directly with a member of academic staff. Online formative assessments provide a safe environment, where trial and error is permitted.

Although online formative assessments such as quizzes and practice examinations are becoming increasingly common in higher education, there is little formal evidence of their educational effectiveness and available data are both contradictory and inconclusive [12]. Nevertheless, such assessments have proved to be popular with pre-clinical medical students [11,14], medical students in clinical attachments [15], students of dentistry [16,17], as well as medical specialist trainees [18]. Thus in modern self-directed medical curricula, formative assessments may be perceived as "a safety net in a self-directed learning course" [5]. However, the potential of these assessments to assist learning by postgraduate trainees has not yet been fully exploited.

We evaluated the impact of online formative assessments on learning by the cohort of students enrolled in the initial 2 years of our undergraduate Medicine program [19] in 2006 and 2007. This employs vertically integrated scenario-based learning for first and second year students during Phase 1, which is comprised of a sequence of nine 8-week courses. Following an introductory Foundations course, these are based on domains related to the life cycle: Beginnings Growth and Development; Health Maintenance; Ageing and Endings; as well as Society and Health. All courses are interdisciplinary: biomedical sciences are integrated with one another; with the social and psychological sciences; and with early clinical experience. Learning is assessed in cross-disciplinary end of course (EOC) written and practical examinations, which are aligned with desired graduate capabilities and support integrated learning [20]. For the purposes of this study, courses in 2006 and 2007 (excluding Foundations, which has no summative assessment) were numbered 1 to 7 in chronological order.

## Methods

### Design and implementation of formative assessments

**Figure 1:**
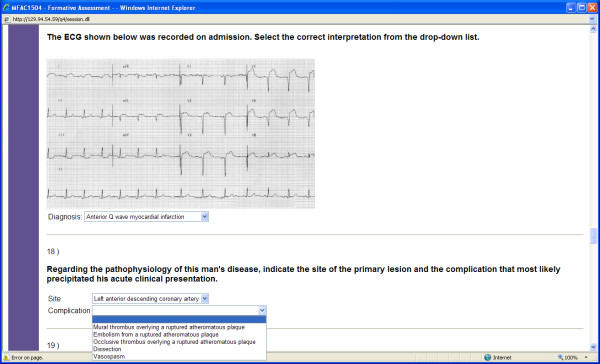
**Screenshot of scenario-based extended matching questions**. Figure 1 shows the use of extended matching questions employing drop-down lists of alternative answers to test the interpretation of diagnostic investigations, as well as correlation with anatomical and pathological concepts underlying coronary artery disease.

**Figure 2:**
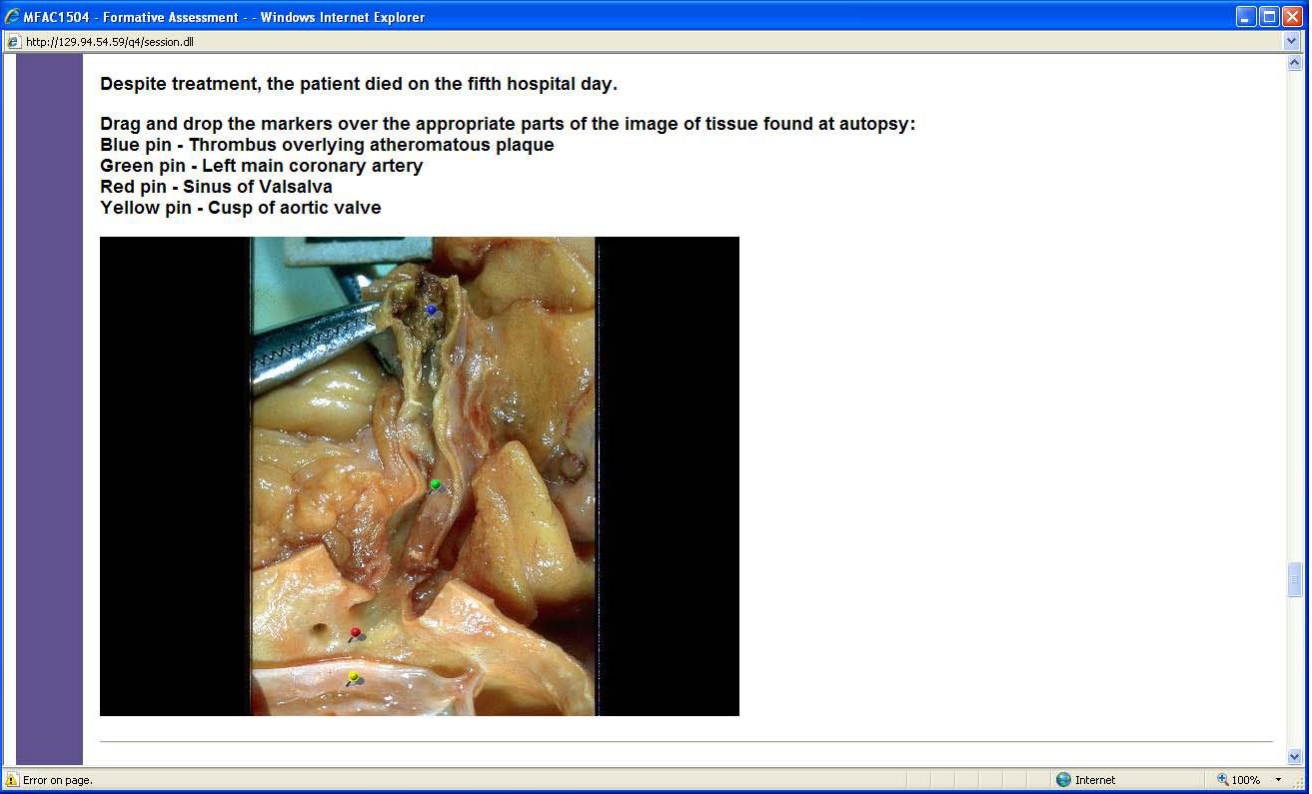
**Screenshot of drag-and-drop question**. Figure 2 shows the use of multiple "hot spots", employing drag and drop markers in an image map to test integrated understanding of Anatomy and Pathology in the context of coronary artery disease.

One of the authors (GMV) developed integrated formative assessments in the biomedical sciences with automated individualized feedback, which were embedded in each of the sequential 8-week courses in the Medicine program, to facilitate take-up by students [21]. There was one formative assessment per course, which was intended to cover material presented throughout that course. Each assessment was made continuously available from week 5 or 6 until the EOC examination in Week 8. The formative assessments were based on clinical scenarios familiar to the students, providing an authentic context for learning, as well as emphasising the curriculum goals of integration between the biomedical sciences and integration of the biomedical with the clinical, social and behavioural sciences. All assessments were peer-reviewed by subject matter experts. The frequent use of image-based questions (Figures 1 and 2) was intended to increase the level of engagement and interactivity for students.

The software tool employed was Questionmark Perception™ (Questionmark, UK). This system is particularly easy to use for developing a wide variety of question types, including drag-and-drop; extended matching (selection and matrix questions); true/false; multiple choice (MCQs); multiple response; as well as text match and short-answer or essay questions. It also provides the capacity to add high-quality graphics, audio or other multimedia to questions and/or feedback. In order to maximize the impact of formative assessments on learning by students, a mix of short answer questions and objective items (primarily MCQs) was employed. This approximates the format of the EOC summative examinations [5], which consist of a combination of four short-answer questions and two blocks of objective items (MCQs), one of which addresses the practical component of the course. However, no questions from the formative assessments were included in the EOC examinations.

Access to the formative assessments was provided via secure links from the university's eLearning website and was made continuously available for several weeks through to the EOC summative examination. Students were able to repeat each formative assessment on multiple occasions if they wished.

### Data collection and statistical analysis

Our study was approved by the Human Research Ethics Committee of The University of New South Wales. Consent for participation in the feedback component of the study was implied by response to the anonymous online survey. Students completed online formative assessments and EOC examinations as part of their learning in Phase 1 of the Medicine program. Correlations between participation and performance in online formative assessments and end of course exams were performed in retrospect. The academic standing of students was not influenced by this study, and students' identities were masked from the investigators in adherence with ethical principles. Therefore, consent was not sought from students.

Participation and performance statistics were gathered via web-based reports from the Questionmark Perception™ results database. Performance in formative assessments was analysed according to each student's best attempt at the assessment. The relationship between these data and EOC marks in seven consecutive Phase 1 courses in 2006 and 2007 was analysed using Student's t-tests, ANOVA and Bonferroni multiple comparisons as appropriate. Regression analyses were used to estimate the component of variation in EOC marks that could be explained by performance in formative assessments. Students who commenced the program in 2004 (n = 8) who were enrolled in Phase 1 in 2006 and for whom data was available, were included in the overall evaluation, but were excluded from the stratified analysis, which focussed on students commencing in 2005 (n = 234), 2006 (n = 235) and 2007 (n = 272).

Student perceptions of the value of online formative assessments were sought via online surveys at the conclusion of the final course in each year, using Likert scales and free-text comments. Comparisons of survey responses between years were performed using a Mann-Whitney U test.

## Results

### Participation and performance

Online formative assessments with automated individualized feedback have proved to be very popular with students – the participation rate for these voluntary assessments has, on average, been greater than 75% in all courses to date. Many students attempted the assessments on multiple occasions, until they achieved mastery of the material. For example, in Course 5 in 2007, of the 466 students who undertook the formative assessment, 233 (50%) made more than one attempt. The mean time students took to complete the formative assessment in each course was 32 ± 5 minutes (range 16 to 50 minutes).

Participation in formative assessments had a statistically significant positive relationship with EOC exam marks in all courses (Table 1). The mean difference in EOC examination marks for those who participated in formative assessments ranged from 6.3% (95% confidence intervals 1.6 to 11.0; p = 0.009) in Course 5 to 3.2% (0.2 to 6.2; p = 0.037) in Course 2 (Table 1). Interestingly, although any attempt at the formative assessments had a positive association with EOC examination marks, we found that repeated attempts at each formative assessment added little benefit (Table 2).

**Table 1:** All students enrolled in Phase 1 in 2006 and 2007 – Effect of participation in online formative assessments on End of Course examination marks

				**End of Course Examination Marks**			
				
**Course**	**≥ 1 attempt**	**N**	**%**	**Mean**	**Mean difference (95% CI)**	**T-Score**	**P value**
**1**	No	99	21.1	60.8	6.0 (3.2, 8.8)	4.2	< .001
				
	Yes	370	78.9	66.8			

**2**	No	62	13.4	62.8	3.2 (0.2, 6.2)	2.1	0.037
				
	Yes	400	86.6	66.0			

**3**	No	70	15.5	66.0	3.5 (1.2, 5.8)	3.0	0.003
				
	Yes	392	84.5	69.5			

**4**	No	90	19.7	59.0	5.5 (3.3, 7.8)	4.3	< .001
				
	Yes	367	80.3	64.5			

**5**	No	44	8.6	59.4	6.3 (1.6, 11.0)	2.7	0.009
				
	Yes	466	91.4	65.7			

**6**	No	71	14.0	59.6	5.3 (2.4, 8.2)	3.6	< .001
				
	Yes	436	86.0	64.9			

**7**	No	122	24.4	61.5	3.6 (1.8, 5.4)	3.9	< .001
				
	Yes	377	75.6	65.1			

Statistical comparisons of End of Course examination marks between students who had attempted the online formative assessment in each course and those who had not were performed by Student's t-tests.

**Table 2:** All students enrolled in Phase 1 in 2006 and 2007 – Relationship between single and multiple attempts at online formative assessments and End of Course examination marks

			**End of Course Examination Marks**					
			
				**ANOVA**		**Multiple comparison**		
			
**Course**	**Attempts**	**N**	**Mean**	**F-score**	**P value**		**Mean difference (95% CI)**	**P value**
**1**	0	99	60.8	13.3	< 0.001	0 VS 1	6.1 (3.2, 9.0)	< 0.001
				
	1	262	66.9			0 VS ≥ 2	5.6 (2.2, 9.1)	< 0.001
				
	≥ 2	108	66.4			1 VS ≥ 2	-.5 (-3.3, 2.3)	1.000

**2**	0	62	62.8	2.2	0.112	0 VS 1	3.1 (-.8, 6.9)	0.166
				
	1	236	65.8			0 VS ≥ 2	3.3 (-.7, 7.4)	0.136
				
	≥ 2	164	66.1			1 VS ≥ 2	.3 (-2.5, 3.0)	1.000

**3**	0	70	66.0	4.8	0.009	0 VS 1	3.1 (.1, 6.1)	0.037
				
	1	227	69.1			0 VS ≥ 2	4.0 (.9, 7.1)	0.007
				
	≥ 2	165	70.0			1 VS ≥ 2	.9 (-1.4, 3.1)	1.000

**4**	0	90	59.0	14.1	< 0.001	0 VS 1	4.5 (1.6, 7.5)	0.001
				
	1	202	63.5			0 VS ≥ 2	6.7 (3.6, 9.7)	0.000
				
	≥ 2	165	65.6			1 VS ≥ 2	2.1 (-.3, 4.5)	0.111

**5**	0	44	59.4	9.1	< 0.001	0 VS 1	7.3 (3.1, 11.6)	< .001
				
	1	233	66.8			0 VS ≥ 2	5.3 (1.0, 9.5)	0.009
				
	≥ 2	233	64.7			1 VS ≥ 2	-2.1 (-4.5, .3)	0.112

**6**	0	71	59.6	6.5	0.002	0 VS 1	5.0 (1.2, 8.7)	0.005
				
	1	246	64.6			0 VS ≥ 2	5.7 (1.8, 9.6)	0.001
				
	≥ 2	190	65.3			1 VS ≥ 2	.7 (-2.0, 3.4)	1.000

**7**	0	122	61.5	7.8	< 0.001	0 VS 1	3.7 (1.4, 6.0)	0.000
				
	1	290	65.2			0 VS ≥ 2	3.0 (.1, 6.0)	0.044
				
	≥ 2	87	64.5			1 VS ≥ 2	.7 (-.3, 1.9)	1.000

Statistical analysis to determine whether a relationship existed between the number of attempts at online formative assessments in each course and End of Course examination marks was performed by ANOVA, with post-hoc testing by Bonferroni multiple comparisons.

For all courses, performance in formative assessments had significant but moderate correlations with EOC examination marks (p < 0.001). The variance in EOC examination marks that could be explained by marks in the formative assessments ranged from 21.8% in Course 6 to 4.1% in Course 7 (Table 3).

**Table 3:** All students enrolled in Phase 1 in 2006 and 2007 – Correlation between formative online assessment score and EOC examination mark

**Course**	**R**	**R^2^**	**B (95% CI)**	**P value**
**1**	.35	.12	.18 (.12, .25)	< .001

**2**	.37	.14	.28 (.19, .37)	< .001

**3**	.42	.18	.23 (.17, .30)	< .001

**4**	.32	.10	.18 (.10, .26)	< .001

**5**	.22	.05	.12 (.07, .17)	< .001

**6**	.47	.22	.29 (.24, .34)	< .001

**7**	.20	.04	.10 (.05, .15)	< .001

Statistical analysis of the relationship between performance in the online formative assessment and EOC examination mark in each course was performed using Pearson's correlation and regression analysis.

### Perceptions

**Figure 3:**
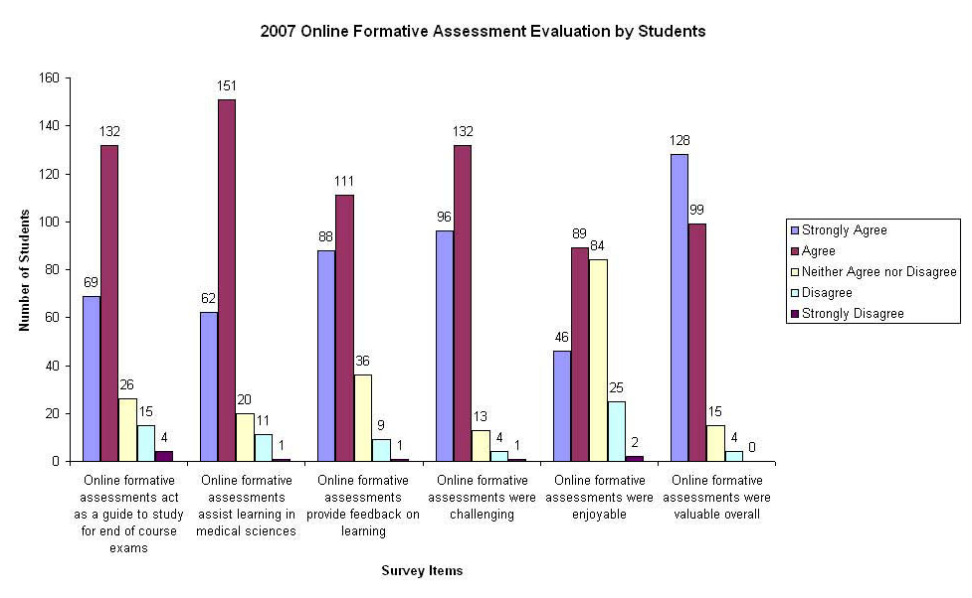
**Student evaluation of online formative assessments in 2007**. The data shown in Figure 3 are based on an online survey with 246 respondents out of 507 students who could potentially have accessed the survey (response rate 48.5%).

**Figure 4:**
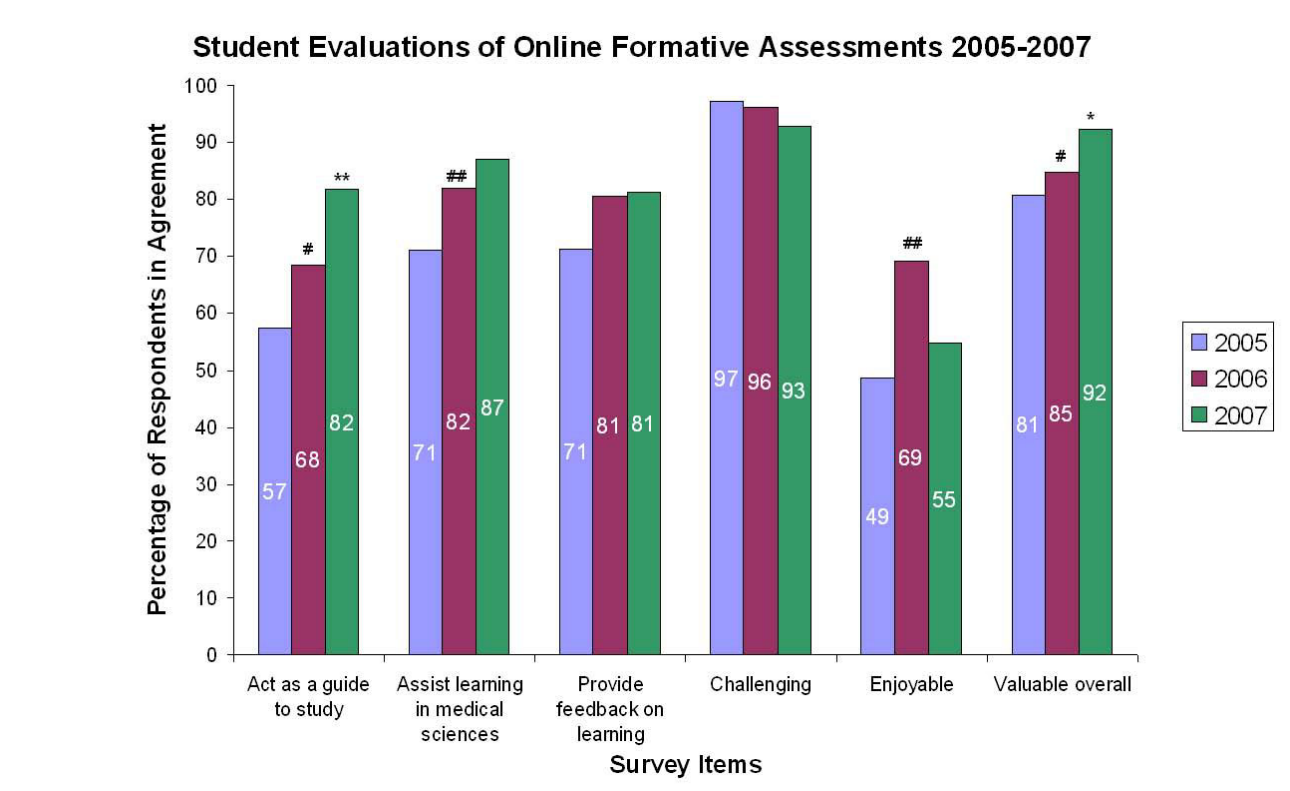
**Comparison of student evaluations of online formative assessments over time**. Figure 4 shows student evaluations of online formative assessments from 2005–2007. Response rates were 52.1%, 51.5% and 48.5% (n = 237, 238 and 246) respectively, based on online surveys. Statistical analysis by Mann-Whitney U tests: # = p < 0.005 compared with 2005 cohort; ## = p < 0.001 compared with 2005 cohort; * = p < 0.005 compared with 2006 cohort; ** = p < 0.001 compared with 2006 cohort.

Student response rates for annual online evaluation surveys from 2005–2007 were 52.1%, 51.5% and 48.5% (n = 237, 238 and 246) respectively. Our response rates (which are underestimates, because they are calculated based on the entire cohort, rather than the number of students who completed the online formative assessment from which the survey was linked) are substantially higher than usually achieved in online course evaluation surveys, and approach those for paper-based surveys [22]. Data from student evaluations (Figures 3 and 4) demonstrated that as well as being challenging and enjoyable, the formative assessments were highly valued by students, both as a means of gaining feedback on learning and in planning their future study. From 2005 to 2007, there have been significant changes in students' positive perceptions of online formative assessments. In 2007, these included significantly higher scores for their utility as a guide to study for the end of course examinations (p < 0.001), as well as their overall value as a learning tool (p < 0.005). Improvements implemented as a consequence of student feedback obtained in 2005 and 2006 are likely to have led to increased positive perceptions by students. Examples of such improvements include: elimination of negative marking for incorrect responses; addition of more short-answer questions (similar to the format of EOC examinations) with suggested marking schemes included in the feedback; whenever possible, making formative assessments available earlier in each course to help guide students' study.

Open-ended feedback comments by students in 2007 provided evidence that the formative assessments were achieving their aims. Relevant examples included assertions that each assessment "provides an opportunity to correct misconceptions and actually learn/relearn concepts." and that there "is integration of a variety of concepts learnt throughout the course", which "gives good insight into students' current performance and areas of potential improvement which can be addressed for the actual exam."

### Year 1 *vs*. Year 2 students in vertically integrated courses

In order to determine whether students benefited more from formative assessments in our vertically integrated medical program during their first or second year, the analysis was stratified according to year of commencement (Table 4). We found no consistent association between the length of time students had been enrolled in the program and the impact on learning of participation in formative assessments. However, there was a tendency for students in the first year of their program to derive greater benefit from participating in formative assessments. For students who commenced in 2006, EOC examination marks were significantly influenced by attempting formative assessments only in their first two courses: Course 1 (mean difference 6.9%; 95% confidence intervals 2.1–11.6; p = 0.006); and Course 2 (6.3%; 1.4–11.1; p = 0.011). Similarly, for students who commenced in 2007, participation in formative assessments significantly influenced EOC examination marks in two out of their first three courses: Course 5 (12.3%; 7.6–17.0; p < 0.001); and Course 7 (4.5%; 2.1–7.0; p < 0.001). It is not clear why first year students in 2007 derived less benefit than second year students from attempting the formative assessment for Course 2. It is plausible that this discrepancy might be explained by the difficulty of the course content, which resulted in an unusually high failure rate, predominantly affecting first year students.

**Table 4:** Analysis of effect of participation in online formative assessments in 2006 and 2007 by year commenced in program

				**End of Course Examination Marks**			
				
**Course**	**Year students commenced in program**	≥ **1 attempt**	**N**	**Mean**	**Mean difference (95% CI)**	**T-Score**	**P value**
**1**	**2005**	No	63	63.8	6.9 (3.6, 10.2)	4.2	< .001
					
		Yes	170	70.7			
	
	**2006**	No	34	56.6	6.9 (2.1, 11.6)	2.9	.006
					
		Yes	200	63.4			

**2**	**2005**	No	41	65.3	3.0 (-.7, 6.7)	1.6	0.114
					
		Yes	188	68.3			
	
	**2006**	No	21	57.8	6.3 (1.4, 11.1)	2.5	.011
					
		Yes	210	64.0			

**3**	**2005**	No	44	67.0	4.5 (1.4, 7.5)	2.9	0.004
					
		Yes	185	71.5			
	
	**2006**	No	25	64.9	2.9 (-.7, 6.4)	1.6	.110
					
		Yes	206	67.8			

**4**	**2005**	No	34	56.7	9.0 (5.6, 12.4)	5.1	< .001
					
		Yes	194	65.7			
	
	**2006**	No	55	60.6	2.5 (-.9, 5.9)	1.5	.148
					
		Yes	173	63.1			

**5**	**2006**	No	23	68.4	2.1 (-1.5, 5.7)	1.2	.242
					
		Yes	205	70.6			
	
	**2007**	No	21	49.5	12.3 (7.6, 17.0)	5.1	< .001
					
		Yes	248	61.9			

**6**	**2006**	No	35	60.6	7.5 (3.5, 11.4)	3.7	< .001
					
		Yes	194	68.1			
	
	**2007**	No	35	59.4	3.4 (-.7, 7.5)	1.6	.107
					
		Yes	228	62.8			

**7**	**2006**	No	56	65.1	2.1 (-.4, 4.6)	1.6	.102
					
		Yes	172	67.2			
	
	**2007**	No	64	59.0	4.5 (2.1, 7.0)	3.7	< .001
					
		Yes	199	63.5			

Statistical comparisons of End of Course examination marks between students who had attempted the online formative assessment in each course and those who had not were performed by Student's t-tests. NB: All courses in Phase 1 of the Medicine program are vertically integrated. Because courses are presented in alternating cycles, students commencing in 2007 had not completed courses 1–4 at the time of data collection, and students who commenced in 2005 completed courses 5–7 in their first year, prior to the period of data collection.

The statistically significant relationship between performance in formative assessments and marks in EOC examinations was maintained regardless of year of commencement in the program (data not shown).

## Discussion

Our data indicate that the online formative assessments in Phase 1 of our undergraduate Medicine program have been effective in promoting learning by students. Students who participated in formative assessments were likely to achieve higher marks in EOC examinations. Better performance in each of the formative assessments was also consistently associated with higher marks in the respective EOC examinations. There was a trend, although not consistent across all courses, for first year students to derive greater benefit from the formative assessments at the commencement of their program, consistent with the notion that such assessments provide a "safety net" for novices in student-centred learning [5].

We believe this report provides much-needed evidence of a quantifiable effect of online formative assessments on learning. Our findings are in contrast to two recent studies in related settings. The first study demonstrated the value of online formative quizzes in improving preparation and participation in classes [23], but reported no effect on summative examination results. The second study was a randomized control trial of online formative assessments for medical students in clinical clerkships, which found no positive effect on learning [24]. It should be noted that the latter investigation, in contrast to our study, failed to gain significant numbers of participating students.

Our findings also contrast with reports suggesting that online formative assessments utilising objective items such as multiple choice questions have no effect on student learning outcomes [23,25], or even a negative effect on learning [26]. The authors of the latter study asserted that multiple choice questions may be unsuitable for formative assessments, because the "lures" or distractors create "false knowledge" [26]. However, these adverse findings were based on the use of multiple choice questions without feedback. In that context, it is not surprising that incorrect answers could be "learned".

Importantly, our results substantially extend the observations of Krasne et al [14], who found that untimed "open-book" formative assessments were good predictors of performance in a summative examination, possibly related to factors such as the reduced pressure compared to a conventional examination format and an emphasis on assessment of higher order learning (e.g. application, evaluation, self-direction). Although our formative assessments employed multiple choice questions (as well as short-answer questions), they were in many respects similar in character to the "open-book" assessments described by Krasne and colleagues [14]. Unlike those assessments, however, ours were integrated across disciplines, broader in their scope, available for a longer period of time, and embedded throughout a program of study. These factors are likely to have increased their efficacy. Furthermore, recent data on the application of test-enhanced learning in higher education [27] validates our use of a combination of short-answer questions and multiple choice questions with immediate feedback in promoting retention of knowledge.

Student perceptions of the formative assessments were uniformly favourable, with consistent and increasingly positive evaluations in online feedback surveys. Correspondingly, there were high participation and repetition rates in the online assessments for each course. The fact that students on average completed the formative assessment within each course in less than one hour might have contributed to the popularity of the assessments, because they were perceived as an efficient means of study for time-poor students.

Our systematic approach to the design, development, implementation and continual improvement of online formative assessments is likely to have played a role in students' perceptions of the assessments, as well as their positive effect on student learning. For example, as part of a continuous improvement cycle, all items used in formative assessments were analysed with regard to difficulty, discrimination co-efficient and correlation with overall assessment outcome. Those that correlated poorly were edited or eliminated from the next cycle, while concepts that proved difficult for students to comprehend were flagged for course conveners.

A limitation of our study is that it is not possible to conclude whether there is a causal relationship between participation and/or performance in online formative assessments and EOC examination marks. It might be that "better" students, who were more highly motivated, were more likely to undertake the formative assessments [12,16]. In our study, multiple attempts at each formative assessment were not associated with higher EOC examination marks. This might suggest that EOC examination performance was primarily influenced by the inherent properties of the students, rather than the salient effects of formative assessment with feedback. Nevertheless, although one reported study has demonstrated no relationship between the effect of online formative assessments and overall student performance in a program as measured by grade point average [16], the design of our study cannot exclude such a relationship. Proving a causal relationship would require a design in which students were randomly assigned to a "control group" within a cohort. This would be inequitable, because a group of students would be deprived of the opportunity to undertake formative assessments during the trial period [12].

### Implications for practice and future research

The results of our study reinforce the impact on learning of well-designed online formative assessments. The highly computer-literate students to whom these assessments were targeted, a population for which web delivery of learning materials and resources is now the default, expressed a very high level of satisfaction. This could in part be related to the graphically intensive approach we used, particularly in visual disciplines such as Anatomy and Pathology. It is likely that this could not be matched by any other mode of delivery. We have evidence from feedback surveys that students pursued further reading and investigated linked resources, so the purpose of provoking further thought about the topics clearly was served.

Thus, while the effort and expense involved in this enterprise has been considerable, this investment is clearly justifiable because the assessments had a high take-up rate and evidently contributed to better learning outcomes for students.

These findings have important implications not only for education of junior medical students but also for continuing education of senior medical students in clinical attachments [15] and especially of junior doctors and specialist trainees. The latter two groups, who are notoriously time-poor, might be attracted to well-packaged formative assessments which they could undertake at their convenience. They might derive considerable benefit from non-threatening feedback on their knowledge and clinical decision-making.

From a research perspective, a question that remains of interest to us is whether the learning benefits of online formative assessments for junior medical students, which we have demonstrated, persist into senior years of medical programs, particularly with respect to understanding of the biomedical sciences. It would also be of interest to determine whether our formative assessments could have diagnostic value, i.e. are those students who perform poorly in formative assessments at their first attempt more likely to fail EOC examinations?

## Conclusion

The results of this study support the contention that well designed formative assessments can have significant positive effects on learning. There is considerable untapped potential for use of formative assessments to assist learning by medical students and postgraduate medical trainees.

## Competing interests

The authors declare that they have no competing interests.

## Authors' contributions

GMV designed, developed and implemented the integrated online formative assessments for medical students described in this study. He was responsible for the study design, data collection and drafting the text of this article and is guarantor. PJ supported the development and implementation of integrated online formative assessments for medical students, as well as editing the text of this article.

HPM supported the development and implementation of integrated online formative assessments for medical students, as well as editing the text of this article. RKK assisted with study design and data analysis, as well as editing the text of this article.

## Pre-publication history

The pre-publication history for this paper can be accessed here:


